# A dataset of clinically recorded radar vital signs with synchronised reference sensor signals

**DOI:** 10.1038/s41597-020-00629-5

**Published:** 2020-09-08

**Authors:** Sven Schellenberger, Kilin Shi, Tobias Steigleder, Anke Malessa, Fabian Michler, Laura Hameyer, Nina Neumann, Fabian Lurz, Robert Weigel, Christoph Ostgathe, Alexander Koelpin

**Affiliations:** 1 Institute of High-Frequency Technology, Hamburg University of Technology, 21073 Hamburg, Germany; 2 Institute for Electronics Engineering, Friedrich-Alexander-Universität Erlangen-Nürnberg (FAU), 91058 Erlangen, Germany; 3 Department of Palliative Medicine, Universitätsklinikum Erlangen, Comprehensive Cancer Center CCC Erlangen - EMN, Friedrich-Alexander-Universität Erlangen-Nürnberg (FAU), 91054 Erlangen, Germany

**Keywords:** Diagnostic markers, Electrical and electronic engineering, Biomedical engineering

## Abstract

Using Radar it is possible to measure vital signs through clothing or a mattress from the distance. This allows for a very comfortable way of continuous monitoring in hospitals or home environments. The dataset presented in this article consists of 24 h of synchronised data from a radar and a reference device. The implemented continuous wave radar system is based on the Six-Port technology and operates at 24 GHz in the ISM band. The reference device simultaneously measures electrocardiogram, impedance cardiogram and non-invasive continuous blood pressure. 30 healthy subjects were measured by physicians according to a predefined protocol. The radar was focused on the chest while the subjects were lying on a tilt table wired to the reference monitoring device. In this manner five scenarios were conducted, the majority of them aimed to trigger hemodynamics and the autonomic nervous system of the subjects. Using the database, algorithms for respiratory or cardiovascular analysis can be developed and a better understanding of the characteristics of the radar-recorded vital signs can be gained.

## Abstract (summary)

**Table:** 

Measurement(s)	vital signs • Respiratory Rate • heart function measurement • Stroke Volume • blood pressure
Technology Type(s)	24 GHz continuous wave radar • thoracic electrical bioimpedance (TEB) • electrocardiography • sphygmomanometry • impedance
Sample Characteristic - Organism	Homo sapiens

Machine-accessible metadata file describing the reported data: 10.6084/m9.figshare.12613109

## Background & Summary

Radar-based detection of vital parameters gains a growing attention in research. The possible applications of contactless and continuous monitoring of heartbeat and respiration are not limited to hospitals^1^, but also include, for example, home care^2^ or driver monitoring in cars^3^. The gold standard methods for monitoring the heartbeat, such as the electrocardiograph (ECG) or the photoplethysmograph, have the disadvantage that they require permanent contact to the skin of a person. This restricts movement or generates false alarms when the electrodes are manipulated, which in turn can lead to alarm fatigue of the personnel^4^. Since clothing or mattresses are transparent to the radar, it can be used in beds, armchairs or car seats to continuously monitor vital signs without touching the person, restricting their movement or reducing their comfort. In the current situation of possible pandemics, in which keeping a minimum distance is an important measure to protect against infection, radar enables short health checks at airports or long-term monitoring of people without having to come into proximity to them. In research there are many approaches to extract the vital parameters of persons from the radar signal. For example, there are groups that use the Fast Fourier transformation, such as Li *et al*.^5^ or Tu *et al*.^6^, to determine the heart and respiratory rate. There are also different approaches that use mode decomposition^7,8^ to obtain the different components of the radar signal. Finally, often the distance signal is processed in the time domain, e.g. by filtering and characterizing the waveform, as shown in^9–12^.

As previously mentioned in^13^, the amount of public data regarding the evaluation of radar-recorded vital signs is very small and so a contribution of data from 11 people has already been made. In this previous work the feasibility of monitoring vital signs using radar in different scenarios was examined. A detailed description of the preclinical study is published in^13^ and the gathered dataset is available at figshare^14^. With the recent completion of our clinical evaluation, a further contribution to a public database shall be made. The previously released database offers 13376 s of data in various scenarios. With the new database, 86459 s of clinically evaluated data will additionally be published. Since a different radar and reference device were used for this study, the data described in this paper need to be viewed separately from those already published. The data were collected by physicians at the university hospital Erlangen according to a defined protocol. The 30 healthy volunteers, two of whom also participated in the previous study, were stimulated in the scenarios using the following methods: the Valsalva manoeuvre (VM), holding breath, and the tilt table test. Furthermore, the Task Force Monitor (TFM) was used as a reference device, which can measure ECG, impedance, impedance cardiogram (ICG) as well as non-invasive continuous blood pressure (BP). Based on these data the TFM determines additional parameters concerning hemodynamics and the autonomic nervous system (ANS). With these synchronized data, reactions after different triggers can be observed and analyzed in the scenarios. Using these data new findings and insights regarding the radar signal can perhaps be gained. A first evaluation of the accuracy of heart and respiratory rates with the radar used was made in^15^.

After the successful completion of this phase of the project GUARDIAN, phase 2, which is currently ongoing, followed. During this second phase, all beds of our partner the palliative care unit of the university hospital in Erlangen were equipped with a radar system under the mattress. With the help of the recorded data, we hope to gain insights into the morphology of the radar signal in terminally ill people and the dying process itself.

## Methods

### Participants

Ahead of planning the experiments, approval was acquired from the local ethics committee. Overall 14 male and 16 female healthy test subjects were measured with an average age of 30.7 ± 9.9 years and an average BMI of 23.2 ± 3.3 kg/m^2^. Before cabling, each test person was informed about the measurements that were conducted and each person was assigned a unique ID for pseudonymisation. During this process, any questions about the radar that arose could be answered. A written consent was obtained from all participants that also allows for sharing the pseudonymised data. All test persons had to fill out a questionnaire on epidemiological data, such as age, sex, weight, and history of diseases. An extract of the data is shown in Table 1. In addition, the condition of the subjects was briefly checked by examining blood pressure, heart rate and heart sound. If all criteria were found to be positive, the subjects could be included in the study and the measurement performed.

**Table 1:** Overview of all test subjects.

ID	Age	Sex^a^	Height (cm)	Weight (kg)	BMI^b^
GDN0001	24	F	166	63	28,9
GDN0002	38	F	161	49	18,7
GDN0003	25	M	187	82	23,4
GDN0004	28	F	178	59	18,6
GDN0005	27	F	173	93	31,1
GDN0006	49	F	172	63	21,3
GDN0007	24	F	187	80	22,9
GDN0008	24	M	182	77	23,2
GDN0009	40	M	184	74	21,9
GDN0010	24	M	186	78	22,5
GDN0011	21	F	165	55	20,2
GDN0012	24	M	193	86	23,1
GDN0013	49	F	173	62	20,7
GDN0014	35	F	153	44	18,8
GDN0015	27	M	182	78	23,5
GDN0016	42	F	165	61	22,4
GDN0017	49	F	167	85	30,5
GDN0018	28	M	165	57	20,9
GDN0019	27	F	166	59	21,4
GDN0020	23	F	172	68	23,0
GDN0021	24	M	187	85	24,3
GDN0022	61	F	178	90	28,4
GDN0023	27	M	186	69	19,9
GDN0024	21	F	165	65	23,9
GDN0025	26	M	183	82	24,5
GDN0026	31	F	160	51	19,7
GDN0027	24	M	187	83	23,7
GDN0028	29	M	190	94	26,0
GDN0029	25	M	186	82	23,7
GDN0030	25	M	172	93	31,4
Mean ± SD^c^	30.7 ± 9.9	—	175.7 ± 10.5	72.2 ± 14.0	23.2 ± 3.3

^a^M: male, F: female, ^b^Body mass index (kg/m^2^), ^c^Standard deviation.

### Human subjects

The study was approved by the ethics committee of the Friedrich-Alexander-Universität Erlangen-Nürnberg (No. 85_15B). All research was performed in accordance with relevant guidelines and regulations. The informed consent was obtained from all subjects in human trials.

### Procedures

**Fig. 1:**
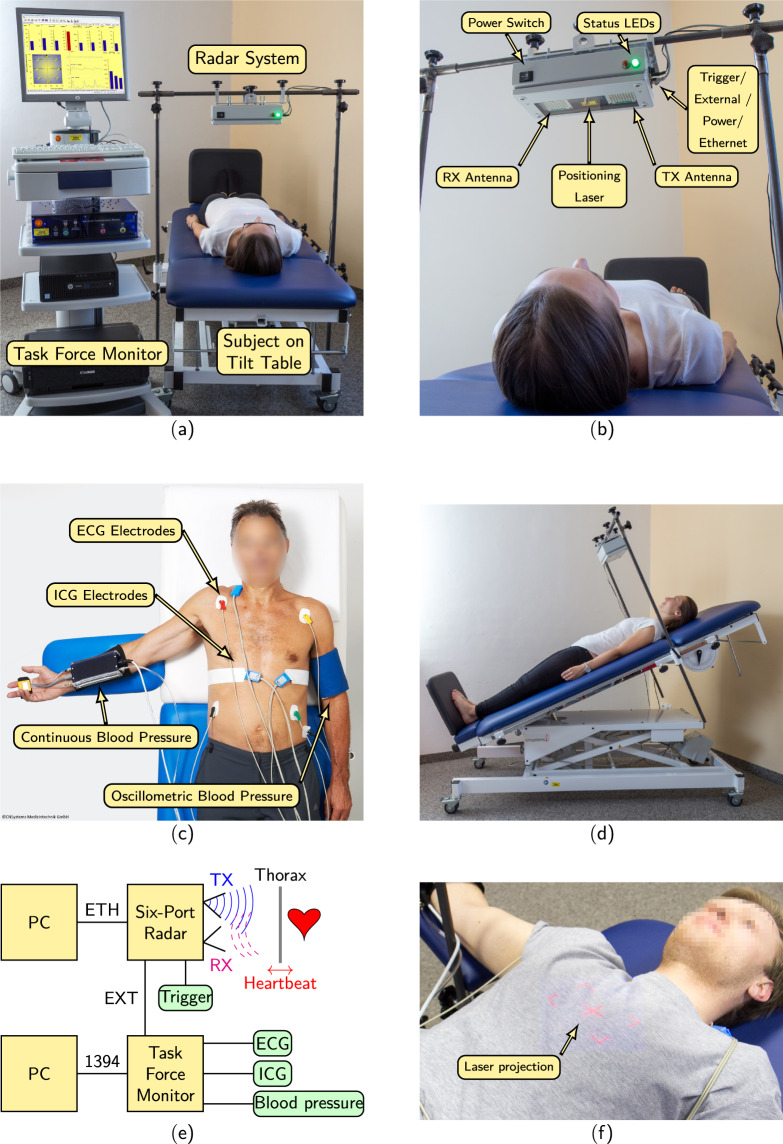
Overview of the measurement setup and the system configuration. (**a**) Photograph of the measurement setup without reference sensor cables. (**b**) Photograph of radar system with descriptions of features. (**c**) Photograph of reference sensors and their locations. (Reprinted under the CC BY license with permission) (**d**) Photograph of the tilt table. (**e**) Block diagram of the system configuration. (**f**) Photograph of the laser projection for positioning.

All measurements were recorded at the Department of Palliative Medicine at the university hospital Erlangen. At least two persons have carried out the measurements. One person was responsible for the protocol, the other person carried out the interventions. After the test persons filled out the questionnaire and gave their consent, the electrodes for the reference measurement were attached to the upper body. The placement of the electrodes can be seen in Fig. 1c. Before the measurement began, the subjects lay down on the tilt table with their upper body facing the radar, as shown in Fig. 1a. Once the BP cuffs and wiring were in place, the subjects were auscultated and the radar systems was moved so that the laser projection was directed at a point where a strong heart sound signal could be perceived. An image of the laser projection is shown in Fig. 1f. Since the focal point between the receiving and transmitting antenna was designed for a distance of around 40 cm, the distance between the radar and the region of interest (ROI) was chosen accordingly during all measurements. A block diagram of the overall setup can be seen in Fig. 1e. In the following, all components will be described in detail.

### Radar system

The radar system used is a further development of the system used in^11^, which was realized with laboratory equipment. It is also based on the Six-Port technology, but has been extended with discrete components into a portable radar system, as can be seen in in Fig. 1a,b,d. In addition, a bistatic antenna design is used to improve signal quality. The inclination angle of the antenna beams is ±10° for transmitting (TX) and receiving (RX) antenna, respectively, with a focal point at 40 cm. The system also has a positioning laser in the middle between the two antennas. This class 1 laser generates a projection on the upper body of the test person, so that the focus point of the system can be easily aligned. The laser in the housing is shown in Fig. 1b and the projection in Fig. 1f. A more detailed view on the system concept of the 24 GHz continuous wave radar is given in^15^. Therefore, the following will only deal with key points of the system to explain the signal processing and vital sign extraction.

The Six-Port structure, on which the system is based, is completely passive and is known to have a high phase resolution^16^. A movement in front of the antenna causes a measurable phase change Δ*φ* between TX and RX signal, which can be converted into a displacement change Δ*x* with the known wavelength λ of the TX signal using^17^:

1 $$\Delta x=\frac{\Delta \varphi }{2{\rm{\pi }}}\cdot \frac{{\rm{\lambda }}}{2}.$$

The raw signals of the radar, called In-Phase (I) and Quadrature (Q) component, are digitized simultaneously using the 24 bit analog-to-digital converter (ADC) *ADS1298* from *Texas Instruments* at a sampling rate of 2000 Hz. The I and Q signal are used to calculate Δ*φ* by arctangent demodulation after a compensation for nonidealities, called ellipse reconstruction, is made. More detailed descriptions can be found in^11^. After sampling the data generated from the ADC are stored in the microcontroller *XMC4500* from *Infineon* that is used to control the radar system. Each time after reaching 50 samples per channel, the data is sent in a UDP packet via Ethernet to a PC for storage and further processing.

Besides controlling the radar, the microcontroller is also used to generate a synchronisation sequence. The sequence, which consists of a sequential binary on and off switching of an analogue pin according to the Gold codes, is sampled simultaneously at the ADC of the radar and is also fed to the external input of the reference system. The processing and synchronisation of the sequence is described in the section “Synchronisation betwwen rader and TFM”. In addition, a push-button is connected to the housing of the radar, which is used during the measurements to set interventions. The button signal is transmitted to another external input of the TFM. An overview of the complete system is given in Fig. 1e. In this representation, the two external signals are combined as EXT, but both are sampled separately at the TFM.

### Reference system

The sensors of the reference system are explained in the paragraphs below. The reference system used in this study is the *Task Force Monitor 3040i* from *CNSystems Medizintechnik GmbH*. The TFM and the corresponding tilt table with the radar mount are illustrated in Fig. 1a. The entire system depicted consists of a monitor, the TFM, a PC, and a printer. In addition, a special tilt table is used which can be tilted up to 90°. In Fig. 1d the table is shown with an angle of about 20°.

In addition to ECG, ICG, oscillometric, and continuous BP the TFM has connections for recording two external inputs. The sensors and external inputs of the system are sampled simultaneously and can be exported from the recording software in a .mat file after the measurement. The included software of the TFM evaluates the raw signals of the sensors during the measurement and also determines different insightful hemodynamic and autonomic parameters of the subject. The sampling frequencies of the individual raw signals described in the following paragraphs refer to the present signals after export from the recording software. Since the signals were processed for synchronisation, other sampling frequencies are provided in the database. The corresponding frequencies are stored additionally in each dataset.

#### ECG

A three channel ECG is used to record electrical activity of the heart during the measurements. The four color coded leads are attached according to clinical standard: red at the right arm, yellow on the left arm, green on the left leg, and black on the right leg. Exemplary applied electrodes are shown in Fig. 1c. A new set of gel electrodes was used for each subject. The TFM recorded the raw data of leads 1 and 2 according to Einthoven’s triangle^18^. In the supplied software lead 3 and the augmented limb leads are calculated from the two raw channels, but not exported to the .mat file. The ECG channels are digitized at the TFM with a sampling rate of 1000 Hz and a precision of ±5 μV.

#### ICG

ICG provides insight into the impedance change of the thorax by applying an alternating small current between two electrodes on the body. Based on Ohm’s law, the measured voltage is proportional to the impedance. Four electrodes are attached to the upper body for the measurement as seen in Fig. 1c: one band electrode on the nape of the neck, two band electrodes lateral on the thorax at the height of the xiphoid process, and one neutral gel spot electrode at the left leg^19^. After export, the ICG raw signal is available with a sampling frequency of 500 Hz, whereas the impedance signal is specified with a sampling frequency of 50 Hz.

#### Blood pressure

The TFM is able to measure a continuous BP signal non-invasively. This technology is called Continuous Noninvasive Arterial Pressure (CNAP) and is done by combining the measurement of an oscillometric BP cuff and a cuff at the fingers measuring the vascular unloading^20^. In Fig. 1c the placement of the BP cuffs is shown. Using this method changes in BP can be monitored during the different interventions. The signal has a sampling frequency of 100 Hz.

#### External input

To enable synchronisation, the external input of the TFM is used. Here the system offers two inputs which are sampled with 1000 Hz. Thus the synchronisation sequence is digitized on one of the inputs and the push-button signal for setting interventions on the other one.

#### Parameters

In addition to the synchronous recording of the various sensors, the TFM software evaluates the individual signals and calculates various parameters of the subject’s hemodynamics and autonomic functions. A list of the parameters with their short and full names is given in Table 2. The values are determined on the basis of each individual heartbeat and are therefore not sampled continuously but with different time intervals beat-to-beat. Because of this, a dedicated time vector is provided for the parameters.

**Table 2:** Overview of aggregated parameters from the reference system. Short names are used in the database.

Short name	Full name	Short name	Full name
SV	Stroke volume	TPR	Total peripheral resistance
CI	Cardiac Index	TPRI	Total peripheral resistance index
HR	Heart rate	dBP	Beat-to-beat dia. blood pressure
HZV	Cardiac output	mBP	Beat-to-beat mean blood pressure
RRI	RR interval	sBP	Beat-to-beat sys. blood pressure
SI	Stroke index	LVET	Left ventricular ejection time
BR	Breathing rate	LVWI	Left cardiac work index
TFC	Thoracic fluid content	LF_HF	Ratio LF_dBP to HF_RRI
ACI	Acceleration index	*_dBP	Different values blood pressure variability
EDI	End diastolic Index	*_RRI	Different values heart rate variability
IC	Velocity index		

### Measurement protocol

The measurements were carried out under the supervision of at least two persons according to an established protocol. Before each scenario, the condition of the test person was queried and the measurements could be stopped by the test person at any time. For some subjects, not all scenarios could be carried out. An *Excel* table, called additional_data.xlsx, with corresponding information is available in the database. The different scenarios are explained in the sections below. After lying down and completing the setup, the subjects were asked to relax for at least 10 min before the measurements were started. During the scenarios the subjects were told to breath calm and avoid large movements.

#### Resting

**Fig. 2:**
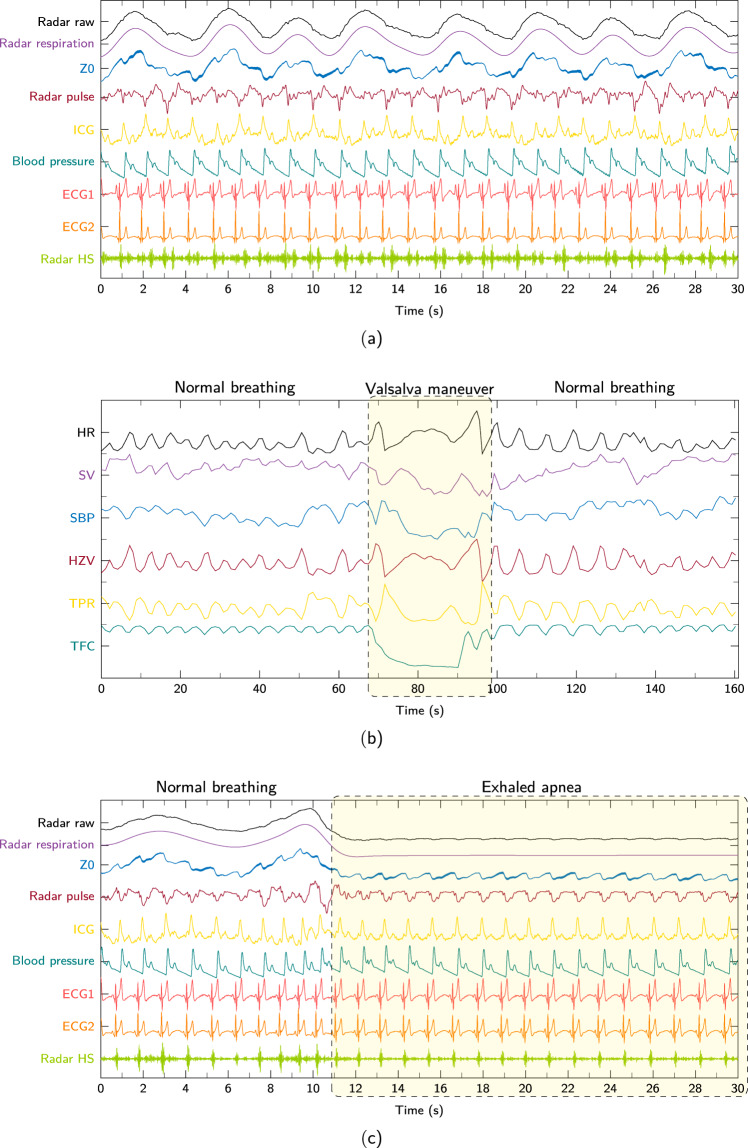
Exemplary signals from radar and TFM during different scenarios: (**a**) Resting, (**b**) Valsalva, and (**c**) Apnea.

**Fig. 3:**
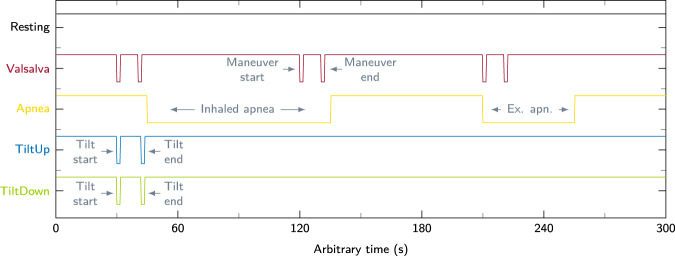
Overview and description of the intervention setting protocol in the different scenarios.

After the relaxation phase the first scenario was started. During the resting scenario the participants continued to lie relaxed with calm breathing. The measurement duration of the scenario is at least 10 min. An exemplary extract of all raw signals from a resting measurement is shown in Fig. 2a. There you can see in the synchronous signals that the test person breathes calmly and it can be derived that the resting heart rate is at about 54 BPM. How to extract the displayed radar vital signs from the raw displacement signal is described in^10,11,15^. Furthermore, in this scenario no marker is set with the push-button as shown in Fig. 3.

#### Valsalva

In the next scenario the Valsalva manoeuvre is performed three times with pauses in between. The manoeuvre is understood to be the forceful expiration against the closed glottis for 20 s^21^. After the VM the test person breathes out and then continues breathing calmly. The sequence of the scenario consists of three runs of 20 s VM with 5 min of recovery in each case afterwards.

The effects of the VM are hemodynamic changes of the circulatory system which are also visible in the parameters measured by the TFM, as can be seen in Fig. 2b. To mark the manoeuvre in the measured data the start and the end is highlighted with the push-button. In Fig. 3 the intervention setting for this scenario is shown as a visual example.

#### Apnea

During this scenario the subjects hold their breath in two defined states as long as possible. In the first state, the subject breathes in completely before apnea, in the second state, the subject breathes out completely before apnea. In Fig. 2c the measured raw signals at the transition from normal respiration to exhaled apnea are illustrated.

The intervention in this scenario is set by the subjects themselves. The test person presses the button as long as the breath is held. This results in the intervention signal seen in Fig. 3.

#### Tilt up

Throughout the tilt up scenario the tilt table is raised to trigger the ANS of the subject. This test leads to a strong reaction of the ANS, among other things, BP and heart rate change significantly. The measurement is started before the table is slowly raised to 70°. Afterwards the measurement is continued for 10 min. While measuring, the start and end of the table movement is marked with the button, as illustrated in Fig. 3.

#### Tilt down

This scenario is the opposite of tilt up. The upright table is moved back to the starting position. Thus, after the start of the measurement, the table is lowered back to 0° and the recording continued for 10. Again reactions of the autonomic nervous system will occur during the procedure. As in the previous scenario, the interventions are set at the start and end of the table movement.

Exemplary signals of three different scenarios can be seen in Fig. 2. In Fig. 2a,c synchronised raw signals of both systems and in Fig. 2b only TFM aggregated parameters are shown. Looking at the raw signal plots you can see the radar displacement signal, radar breathing, impedance, radar pulse, ICG, BP, both ECG signals, and radar heart sound. Apart from the radar vital parameters only the ECG signals were bandpass filtered in the range of 1 Hz to 20 Hz.

## Data Records

The radar measurements were recorded using *MathWorks MATLAB* and since the TFM recording software also allows a corresponding export, the data were saved as .mat files. All datasets are available online at *figshare*^22^ (10.6084/m9.figshare.12186516). Furthermore, an auxiliary table, called additional_data.xlsx, is available from figshare in which an overview of the database and subjects is given.

The structure of the database is fairly simple. The measurement data of each subject are stored in a separate folder with the name as the persons ID. In each folder, there is one .mat file for each scenario that the subject performed. Every .mat file has the same structure and consists of different vectors representing the raw signals. Besides the signals there are also variables for the sampling frequencies of each signal and a struct with the TFM parameters. radar_I and radar_Q are the I and Q signals of the radar system with which the relative distance can be determined. Both signals are stored in mV and the corresponding sampling frequency is called fs_radar. All signals coming from the TFM have the prefix tfm_. Thus tfm_ecg1 and tfm_ecg2 represent the two ECG signals specified in mV. Furthermore, there is the ICG tfm_icg which is given in Ω/s, the impedance signal tfm_z0 which is given in Ω, the BP signal tfm_bp which is given in mmHg, and the intervention signal tfm_intervention which given in V. Then there is the struct called tfm_param with the parameters calculated by the TFM. Table 2 gives an overview of all the parameters stored in the struct but there is also a sheet in the “additional_data” table with information about the corresponding units. At last there is a cell array called measurement_info containing three values: The timestamp of recording, the scenario and the subject ID.

The “additional_data” table is separated in four sheets called “subject overview”, “scenario durations”, “measurement info”, and “TFM parameters”. In the first sheet an overview of the epidemiological data of the subjects is given. Next, the duration of each scenario split for each subject is listed and the third sheet explains missing data for each person and scenario, if applicable. Finally, the parameters of the TFM are listed with short and full names as well as their units.

Table 3 shows the recording duration of the scenarios split by subject available in the database. For each scenario and each test person the total length in seconds as well as the sum of the durations is displayed. Thus it is noticeable that not every test person has gone through the entire protocol. However, the resting scenario was carried out with each subject and so an average recording time of 2882 s per subject is obtained. This results in a total recording time of 86459 s, which corresponds to about 24 h of clinically recorded reference synchronised radar data.

**Table 3:** Overview of all test subjects showing the duration per scenario in seconds.

ID	Resting (s)	Valsalva (s)	Apnea (s)	TiltUp (s)	TiltDown (s)	Total (s)
GDN0001	607,6	1000,5	0,0	98,2	684,7	2390,9
GDN0002	622,4	993,5	0,0	589,0	417,0	2621,8
GDN0003	601,4	1057,1	0,0	768,7	642,8	3069,9
GDN0004	603,1	1088,3	146,9	697,6	664,1	3199,9
GDN0005	610,1	1083,0	150,9	448,1	605,3	2897,3
GDN0006	610,9	1309,6	143,7	795,7	665,7	3525,5
GDN0007	634,9	991,5	186,3	634,9	644,7	3092,4
GDN0008	618,6	987,6	106,9	707,0	660,4	3080,4
GDN0009	649,4	976,7	227,4	641,9	631,7	3127,1
GDN0010	639,1	991,7	264,8	642,9	638,0	3176,5
GDN0011	648,9	976,0	204,9	633,4	629,0	3092,2
GDN0012	648,5	980,3	401,8	636,1	635,4	3302,1
GDN0013	725,6	1009,5	108,9	661,3	642,7	3148,0
GDN0014	603,5	983,5	394,2	654,9	631,5	3267,6
GDN0015	648,5	0,0	0,0	0,0	0,0	648,5
GDN0016	610,6	991,4	113,4	709,6	682,6	3107,5
GDN0017	603,3	1060,4	152,9	644,7	652,0	3113,2
GDN0018	636,0	1159,1	95,4	0,0	0,0	1890,5
GDN0019	603,1	1105,0	124,3	682,1	666,7	3181,1
GDN0020	640,2	987,0	246,8	718,1	599,4	3191,3
GDN0021	613,9	1066,5	104,6	760,3	637,0	3182,3
GDN0022	659,8	1094,9	153,7	650,2	677,7	3236,3
GDN0023	678,9	992,4	291,7	655,6	640,8	3259,3
GDN0024	610,5	0,0	0,0	0,0	0,0	610,5
GDN0025	616,9	1028,2	341,7	648,1	640,6	3275,5
GDN0026	821,0	0,0	0,0	694,6	767,3	2282,9
GDN0027	627,9	1037,3	167,7	669,5	665,5	3167,8
GDN0028	611,9	992,7	242,2	635,9	637,5	3120,0
GDN0029	615,9	996,5	165,5	645,4	641,1	3064,3
GDN0030	626,6	1028,5	169,0	656,8	655,8	3136,7
Total (s)	19048,6	27968,3	4705,2	17380,2	17356,8	86459,0

## Technical Validation

**Fig. 4:**
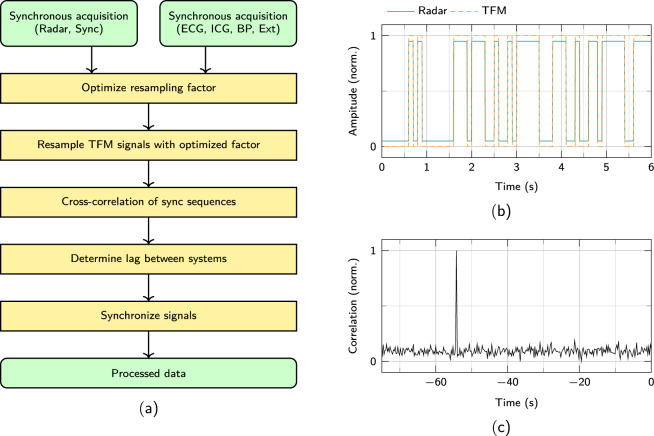
(**a**) A flowchart showing the signal acquisition and synchronisation process. (**b**) The synchronisation sequences recorded of both systems aligned after lag removal. (**c**) Cross-correlation of the synchronisation sequences. The position of the maximum corresponds to the lag between radar system and TFM.

The entire data processing to obtain the synchronised data of the published database is shown in Fig. 4a. After a test person has been recorded according to the protocol, the data of both systems are gathered. In a first step, these signals are combined using an automatic synchronisation process. Afterwards, the synchronised data are saved in one file.

### Synchronisation between radar and TFM

Since the radar and the TFM sample their data asynchronously from each other, the data must be synchronised during post processing. Therefore, a synchronisation sequence is generated in the microcontroller of the radar, which is sampled both by the ADC of the radar and at the external input of the TFM. In Fig. 4b the sampled sequences of both recordings are shown after removing the time lag between the systems.

The Gold codes were selected as a binary sequence for synchronisation. These have the property that the cross-correlation of different sequences is minimal. Only the cross-correlation with the same sequence results in a high correlation value^23^.

However, since both ADCs operate at a different sampling frequency, the recorded signal of the TFM must be upsampled before correlation. The sampling frequencies of the TFM and the radar are 1000 Hz and 2000 Hz according to the datasheets. However, if the real sampling frequencies of the systems differ even slightly from the datasheet, resampling with a simple factor of two would distort the signals. Therefore, an optimization procedure is used to determine a resampling factor as exact as possible. The resampling factor is adjusted automatically using an optimization method until the peak of the correlation becomes maximized. The factor at which the maximum of the resulting cross-correlation is at its highest is chosen. The factor is then used to resample all raw signals of the TFM to compensate for the deviation between the two sampling frequencies. For this reason, the sampling frequencies of the raw signals of the TFM are twice as high in the database as mentioned in the Section “Measurement protocol”.

An exemplary cross-correlation of two recorded sequences is pictured in Fig. 4c. There you can see a maximum at about −54 s. This means that the radar signal is shifted by 54 s with regard to the TFM signals. The resulting time lag is then corrected accordingly and the synchronous regions are stored. Since there is no further information in the sync sequence it is not saved in the combined dataset.

### Correlation of radar and ECG interbeat-intervals

In order to technically validate the data, the interbeat-intervals (IBIs) of the radar heart sound signal shall be correlated with those of the reference signal. Therefore, the successive heartbeats in the individual signals must be detected and their time difference calculated. In case of the reference ECG signal this means to detect the R-peaks, the locations where the heartbeats occur. For this purpose the algorithm from Zhang^24^ is used. The IBIs of the ECG are then calculated from the determined R-peak locations. To be able to determine the intervals from the heart sound signal, it must be segmented. An algorithm based on hidden semi-Markov models from^25^ is used for segmentation. This algorithm assigns the following segments according to the heart cycle to the sound signal: first heart sound (S1), systole, second heart sound, and diastole. Since the timing of the S1 corresponds to the R-peak, the start of the S1 is taken as the location of the heartbeat in the sound signal. With this information the IBIs can be calculated from the radar signal.

**Fig. 5:**
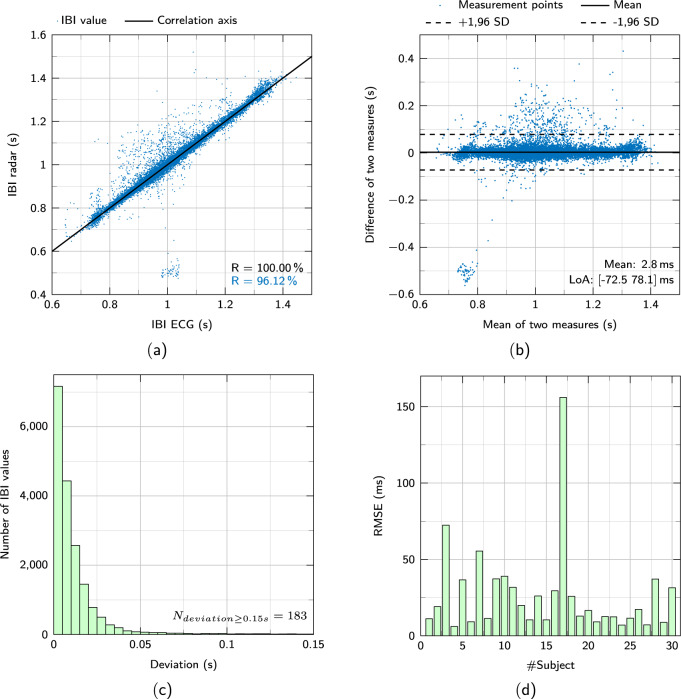
Scatter diagram (**a**) and Bland-Altman (**b**) plot of all IBI values. (**c**) Histogram showing the deviation of all IBI values from their reference. (**d**) The RMSE of the resting scenario seperated by subjects.

In order to compare the IBI values from both signals, the values need to be sampled equidistant and at the same time points. Therefore, the values are resampled with the sampling frequency of the radar and afterwards, IBI values are taken at identical and equidistant intervals of one second. The correlation of the resampled IBIs of the resting scenario data is illustrated in Fig. 5a. In black the correlation axis with a perfect value of 100% is drawn. The majority of the IBI values hardly deviate from this axis, resulting in a very high Pearson correlation coefficient of R = 96.12% (p < 0.001). This small deviation can also be seen in the histogram in Fig. 5c. In this diagram, the number of IBI values is plotted against their deviation from the reference. Accordingly, 11598 values of the total 18297, which corresponds to about 63.4%, deviate less than 10 ms from the reference value and 96% of the values deviate less than 50 ms.

A Bland-Altman representation to analyze the IBI values is given in Fig. 5b. The mean difference is 2.8 ms as indicated by the solid black line and the lower and upper limits of agreement, indicated as dashed black lines, are −72.5 ms and 78.1 ms, respectively. Furthermore, no trend can be seen in the interval. At last, in Fig. 5d the root mean square error (RMSE) of the IBI deviations is shown separated by subject. For the majority of the test persons the RMSE is below 25 ms in the resting scenario, only test person 17 has a higher deviation. This can be explained due to a bad measurement result and an incorrectly selected measuring point.

## Usage Notes

The whole dataset is freely available at *figshare*. All the records are stored in .mat format and can be analyzed in *MATLAB*. Due to size constrains the dataset is split in three zip files with each containing 10 subjects. Download the files and unzip them in one folder using a tool like *7zip*. These files can of course also be converted into different formats to use with other software applications.

As described in the Section “Radar system”, the distance signal can easily be reconstructed from *I* (radar_I) and *Q* (radar_Q) using a simple arctangent demodulation. However, due to nonidealities at the front end, amplitude and phase imbalances occur inside the Six-Port structure. This leads to offset, gain, and phase errors within the sampled signals. Before the demodulation can be applied, an ellipse fitting algorithm such as presented in^26^ has to be employed. Further descriptions can be taken from^11^. An example is also given in the code samples which are available online.

## Data Availability

The entire code used for the technical validation is available from https://gitlab.com/sven_schellenberger/scidata_phase1. Furthermore, a script for viewing the data is also included in the repository which can easily be used by configuring the subject ID and scenarios which shall be viewed. The code was written and tested using *MATLAB* R2020a for *Microsoft Windows*.
